# Efficacy of 2-Hydroxyflavanone in Rodent Models of Pain and Inflammation: Involvement of Opioidergic and GABAergic Anti-Nociceptive Mechanisms

**DOI:** 10.3390/molecules27175431

**Published:** 2022-08-25

**Authors:** Faiz Ali Khan, Gowhar Ali, Khista Rahman, Yahya Khan, Muhammad Ayaz, Osama F. Mosa, Asif Nawaz, Syed Shams ul Hassan, Simona Bungau

**Affiliations:** 1 Department of Pharmacy, University of Peshawar, Peshawar 25000, Pakistan; 2 Department of Pharmacy, Faculty of Biological Sciences, University of Malakand, Chakdara 18000, Pakistan; 3 Public Health Department, Health Sciences College at Lieth, Umm Al Qura University, Makkah 24231, Saudi Arabia; 4 Biochemistry Department, Bukhara State Medical Institute Named after Abu Ali Ibn Sino, Bukhara 281403, Uzbekistan; 5 Shanghai Key Laboratory for Molecular Engineering of Chiral Drugs, School of Pharmacy, Shanghai Jiao Tong University, Shanghai 200240, China; 6 Department of Natural Product Chemistry, School of Pharmacy, Shanghai Jiao Tong University, Shanghai 200240, China; 7 Department of Pharmacy, Faculty of Medicine and Pharmacy, University of Oradea, 410028 Oradea, Romania

**Keywords:** analgesia, neuropathy, GABAergic mechanisms, allodynia, hyperalgesia

## Abstract

The current work examined the pharmacological potential of a selected flavanone derivative 2-hydroxyflavanone as a promising remedy for the treatment and management of pain. The selected flavanone derivative (2-HF) was evaluated for its analgesic and anti-inflammatory potentials following standard pharmacological protocols including hot plate, acetic acid-induced writhing and tail immersion tests. Naloxone and pentylenetetrazol were used to evaluate the potential implication of GABAergic and opioidergic mechanisms. The anti-inflammatory potential of 2-HF was confirmed using carrageenan-, serotonin- and histamine-induced paw edema models as well as a xylene-induced ear edema model. Furthermore, the anti-neuropathic potential of 2-HF was tested using a cisplatin-induced neuropathic pain model. Our sample, at the tested concentrations of 15, 30 and 45 mg kg^−1^, showed considerable analgesic, anti-inflammatory effects, as well as efficacy against neuropathic pain. Naloxone and pentylenetetrazol at 1 and 15 mg kg^−1^ antagonized the anti-nociceptive activities of 2-hydroxyflavanone indicating the involvement of opioidergic and GABAergic mechanisms. In the static allodynia model, combination of gabapentin 75 mg kg^−1^ with 2-HF at 15, 30, 45 mg kg^−1^ doses exhibited considerable efficacy. In cold allodynia, 2-hydroxyflavanone, at doses of 15, 30 and 45 mg kg^−1^ and in combination with gabapentin (75 mg kg^−1^), demonstrated prominent anti-allodynic effects. The paw withdrawal latency was considerably increased in gabapentin + cisplatin treated groups. Moreover, cisplatin + 2-hydroxyflavanone 15, 30, 45 mg kg^−1^ showed increases in paw withdrawal latency. Likewise, considerable efficacy was observed for 2-hydroxyflavanone in thermal hyperalgesia and dynamic allodynia models. Our findings suggest that 2-hydroxyflavanone is a potential remedy for pain syndrome, possibly mediated through opioidergic and GABAergic mechanisms.

## 1. Introduction

Therapy of pain remains a major medical problem, as millions of patients need medical attention in order to obtain medication each year to alleviate their pain [1]. The International Association of Pain has described pain as an unpleasant sensory and emotional sensation accompanied by damage to tissue [2]. Based on the duration, the pain might be grouped as an acute or a chronic experience. Acute pain functions as protection and is the bodies response to tissue damage. Normally, nociceptive pain lasts for a short time and disappears upon healing. Unlike acute pain, chronic pain is an unusual perception in response to direct damage or injury to nerves. Chronic pain experienced by individuals involves neuropathic and inflammatory pain [3,4]. Neuropathic pain is usually characterized by diverse etiologies which involve inflammation, viral infection, metabolic disorder, trauma and irradiation- or chemically-induced nerve damage [5,6].

For the treatment of pain, patients are given medication on the basis of the type of pain experienced they are experiencing. For acute pain treatment, opioid analgesics are generally used which act mainly by affecting µ-opioid receptors. However, NSAIDs are preferred for the management of inflammatory pain. These act by inhibiting the cyclooxygenase (COX) enzyme, leading to inhibition of prostaglandins synthesis. However, adverse side effects can be caused by both opioid analgesics and NSAIDs [7]. Local anesthetics are another significant group of pain-alleviating drugs. Local anesthetics block conduction of nerves by changing the function of cross membrane voltage-gated Na-channels. Membrane voltage-gated sodium channels are therefore effective analgesics which prevent the transmission of painful sensations to the central nervous system (CNS). Currently, antiepileptic agents and local anesthetics are utilized for neuropathic pain syndrome treatment and just a small number of drugs for pain treatment have been confirmed by the Food and Drug Administration (FDA) [8]. These therapeutic agents are utilized for pain treatment but a large number of patients still do not attain complete relief from pain. Hence, there is an urgent medical need to identify new safe and effective therapeutic agents beyond the presently accessible analgesic agents [9,10].

Flavonoids are a well-defined class of polyphenolic synthetic or natural compounds [11,12], usually found in wine, vegetables, fruits, cereals and tea and have considerable pharmacological activities such as antioxidant [13,14], anticancer [15,16,17,18], anti-Alzheimer [19,20], anti-inflammatory [21,22,23] and anxiolytic [24]. Flavonoids and their derivatives have been extensively studied for their beneficial analgesic effects. One chemically altered form of flavonoid is 2-Hydroxyflavanone, which is responsible for bioactivity and bioavailability. Because of the diverse pharmacological activities of flavanones, it is presumed that our selected flavanone derivative, 2-hydroxyflavanone, may be a promising solution for the management of pain. Keeping in view the pharmacological potentials of flavonoids and their derivatives, the current study aimed to evaluate the analgesic, anti-inflammatory potentials of 2-HF and appraise its efficacy in neuropathic pain using standard models.

## 2. Results

### 2.1. Effect of 2-HF in Hot Plate Test

**Figure 1:**
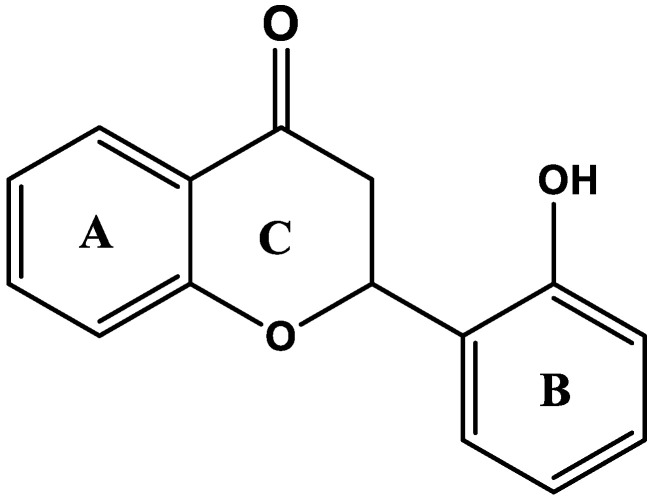
Chemical structure of 2-hydroxyflavanone.

**Figure 2:**
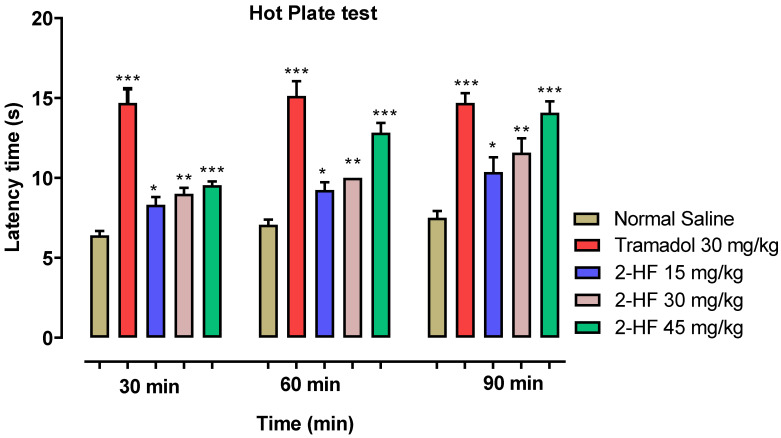
Results of hotplate study performed on 2-HF. *** *p* < 0.001, ** *p* < 0.01, * *p* < 0.05 when compared with control, two-way repeated measures ANOVA followed by Tukey’s post hoc test.

The thermal nociceptive potential of the test compound (Figure 1) was determined by holding the animals over a hot plate until a nociceptive response was observed such as jumping, flicking or licking of hind limbs. For the saline group, latency time was 6.42 ± 0.27, 7.06 ± 0.32 and 7.5 ± 0.42 s after 30, 60 and 90 min respectively. The group treated with 2-hydroxyflavanone (2-HF) at 15 mg kg^−1^ after 30, 60 and 90 min, showed an increased latency time of 8.3 ± 0.5, 9.25 ± 0.48 and 10.36 ± 0.92 s respectively as compared with saline control. Treated groups of 2-HF at 30 and 45 mg kg^−1^ dose exhibited a gradual rise in the latency time in comparison to saline treated group, The latency time for the treated group of 2-HF (30 mg kg^−1^) was 9.01 ± 0.37, 10.04 ± 0.22 and 11.5 ± 0.90, seconds whereas the latency time for treated group of 2-HF (45 mg kg^−1^) verses saline control group was 9.5 ± 0.23, 12.8 ± 0.6 and 14.08 ± 0.7 s. Tramadol, being a centrally acting analgesic agent, was used to confirm the involvement of central anti-nociceptive mechanisms in the efficacy of 2-HF. For the tramadol treated group (30 mg kg^−1^), the latency time after 30, 60 and 90 min was observed as 14.7 ± 0.87, 15.1 ± 0.9 and 14.08 ± 0.42 s respectively as displayed in Figure 2.

### 2.2. Opioidergic and GABAergic Mechanism of 2-HF

**Figure 3:**
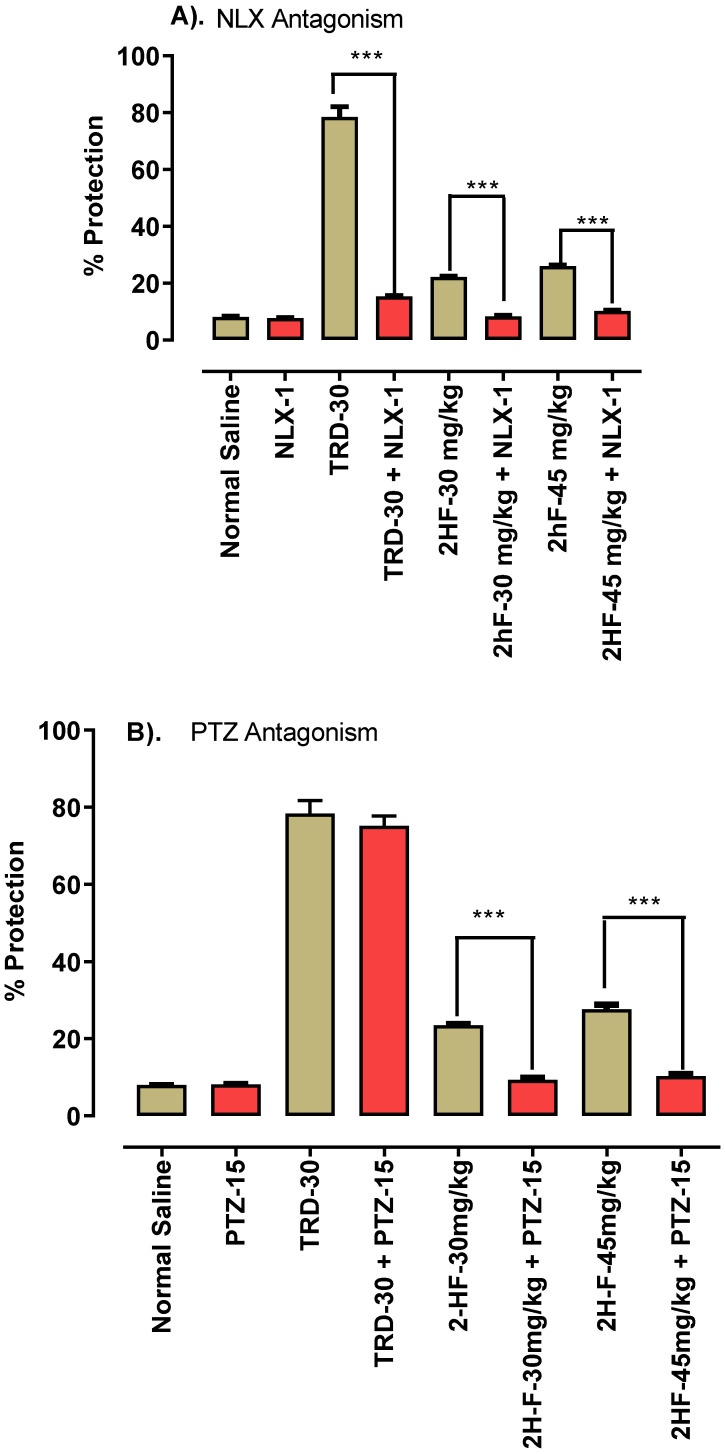
(**A**) Naloxone effect at 1 mg kg^−1^ and (**B**) PTZ effect at 15 mg kg^−1^. *** *p* < 0.001 when compared with control.

Naloxone (NLX) and pentylenetetrazol (PTZ) were used as antagonists for assessment of the anti-nociceptive mechanisms of 2-HF. The analgesic potential of 2HF using hot plate apparatus at 30 mg kg^−1^ dose was antagonized by 1 mg kg^−1^ naloxone. Likewise, at 45 mg kg^−1^ dose of 2-HF, the analgesic potential was antagonized by 1 mg kg^−1^ naloxone as a higher withdrawal latency of paw in seconds was noted, suggesting an opioidergic mechanism for 2-HF. The analgesic action of tramadol (30 mg kg^−1^) was antagonized by administration of naloxone as displayed in Figure 3A. In a hot plate apparatus, the analgesic action of 2-HF (30 mg kg^−1^) was reduced by pentylenetetrazol at a dose of 15 mg kg^−1^. Similarly, antagonizing potential for 2-HF was recorded when pentylenetetrazol 15 mg kg^−1^ was administered to the 2-HF (45 mg kg^−1^) treated group, suggesting the involvement of a GABAergic mechanism for 2-HF as shown in Figure 3B.

### 2.3. 2-HF Reduce Acetic Acid-Induced Writhing in Animals

**Figure 4:**
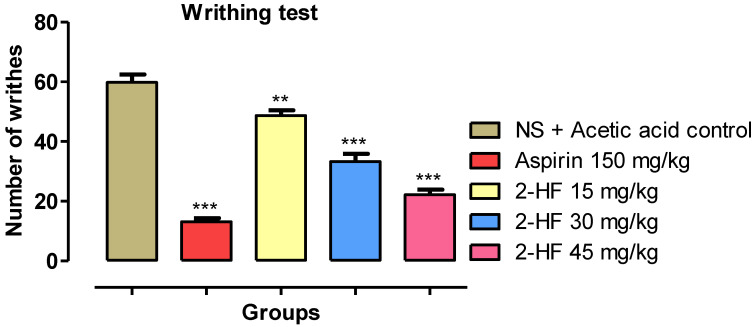
Result of 2HF in writhing test. *** *p* < 0.001, ** *p* < 0.01, * *p* < 0.05 versus disease control.

Acetic acid injection was accompanied by an enormous rise in the number of writhing. Saline control group demonstrated 59.83 ± 2.64 number of writhing, whereas, in comparison to saline group, the number of writhing by 2-HF at 15, 30 and 45 mg kg^−1^ was decreased to 48.66 ± 1.84, 33.33 ± 2.55 and 22.16 ± 1.72 respectively. The number of writhing by the positive control anti-inflammatory agent aspirin (150 mg kg^−1^) treated group decreased significantly to 13.16 ± 1.19 as demonstrated in Figure 4.

### 2.4. Analgesic Study Using Tail Immersion Paradigm

**Figure 5:**
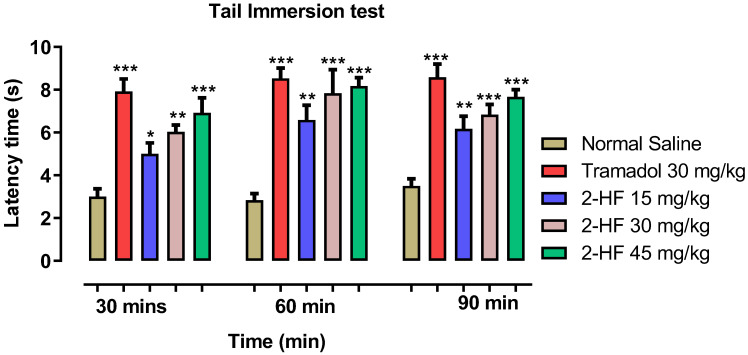
Results of 2HF in tail immersion model. *** *p* < 0.001, ** *p* < 0.01, * *p* < 0.05 vs. control, two-way repeated measures ANOVA followed by Tukey’s post hoc test.

Our tested compound (2-HF) demonstrated a marked enhancement in the tail withdrawal latency time versus saline control group. A rise in the withdrawal latency by the 2-HF (15 mg kg^−1^) treated group was noted i.e., 5 ± 0.51 s versus 3 ± 0.36 s for the saline control group for 30 min, while at 60 min an increase to 6.58 s was noted and at 90 min a reduction in latency time was noted, which was 6.16 s over the time. The 2-HF at 30 mg kg^−1^ treated group revealed a rise in latency time of 6.003 ± 0.31 s and 2-HF (45 mg kg^−1^) showed 6.9 ± 0.71 s versus 3.02 ± 0.30 s for the saline control group at 30 min and 7.8 s ± 1.1 and 8.6 ± 0.401 at 60 min. However, latency time generally decreased at 90 min. The tramadol treated group (centrally acting positive control) at 30 mg kg^−1^ revealed a significant response of 7.9 ± 0.58 s at 30 min, 8.53 ± 0.48 s at 60 min and 8.58 ± 0.61 s at 90 min versus control as demonstrated in Figure 5.

### 2.5. 2-HF Reduce Carrageenan, Serotonin and Histamine-Induced Inflammation

**Figure 6:**
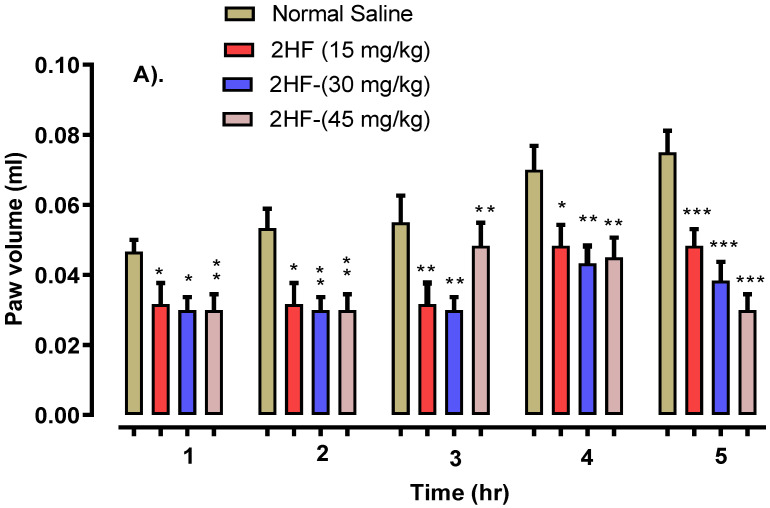

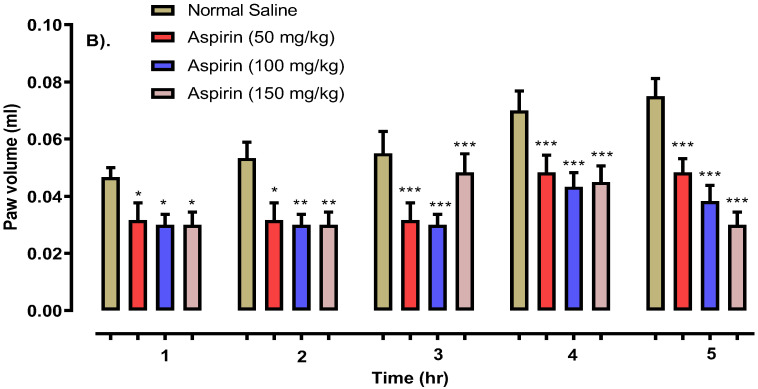
Anti-inflammatory effect of 2-Hydroxyflavanone using carrageenan-induced paw edema model. (**A**). Anti-inflammatory activity of 2HF at 15, 30 and 35 mg/kg dose. (**B**). Anti-inflammatory activity of Aspirin at 50, 100 and 150 mg/kg dose. *** *p* < 0.001, ** *p* < 0.01 and * *p* < 0.05 in comparison to control group, two-way repeated measures ANOVA followed by Tukey’s post hoc test.

Our tested compound (2-HF) produced an anti-inflammatory, dose-dependent effect using carrageenan and serotonin as well as histamine-induced paw edema models. Animals treated with 15 mg kg^−1^ of 2-HF decreased the temporal inflammatory effect induced by carrageenan i.e., *p* < 0.05, after 1 h, *p* < 0.01 after 3 h and *p* < 0.001 after 5 h as shown in Figure 6.

**Figure 7:**
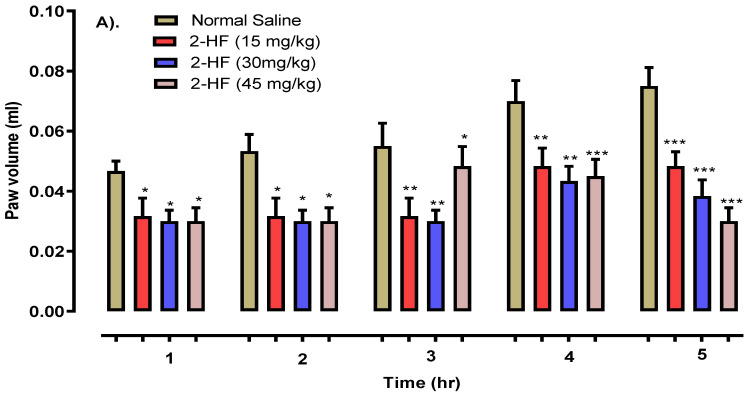

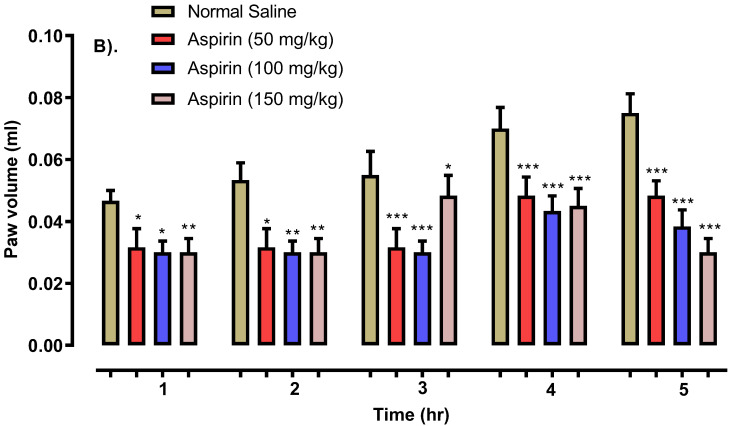
Results of the anti-inflammatory potentials of 2-HF in histamine-induced paw edema model. (**A**). Anti-inflammatory activity of 2HF at 15, 30 and 35 mg/kg dose. (**B**). Anti-inflammatory activity of Aspirin at 50, 100 and 150 mg/kg dose. *** *p* < 0.001, ** *p* < 0.01, * *p* < 0.05 in comparison to saline treated group, two-way repeated measures ANOVA followed by Tukey’s post hoc test.

**Figure 8:**
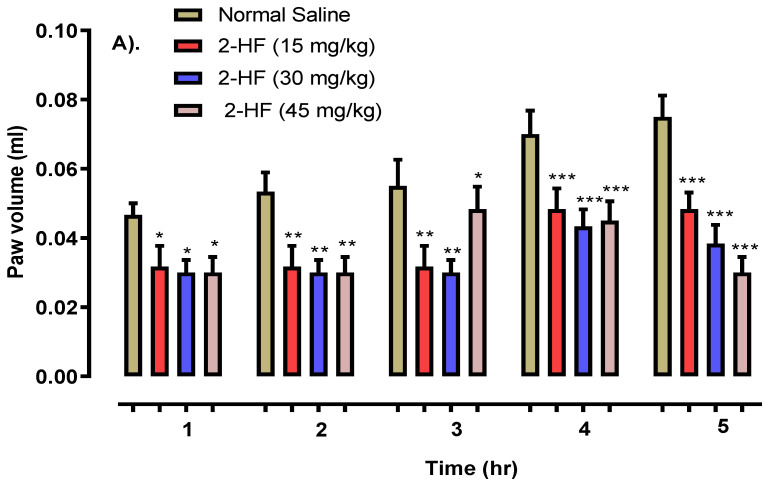

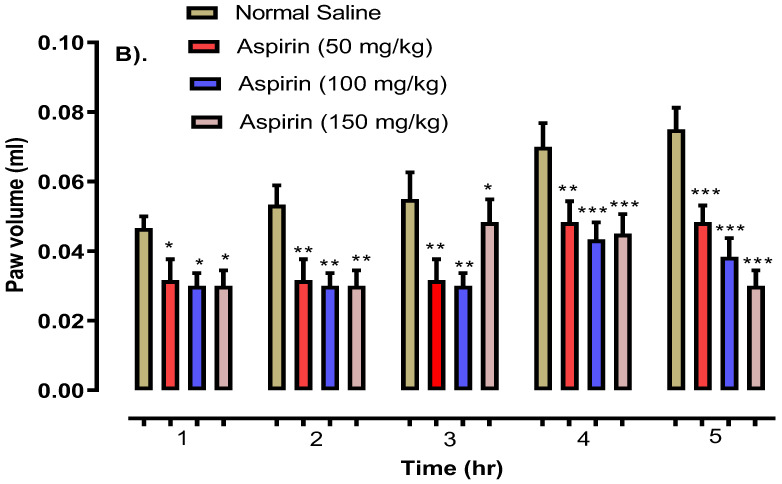
Results of the anti-inflammatory study using the serotonin-mediated paw oedema model. (**A**). Anti-inflammatory activity of 2HF at 15, 30 and 35 mg/kg dose. (**B**). Anti-inflammatory activity of Aspirin at 50, 100 and 150 mg/kg dose. *** *p* < 0.001, ** *p* < 0.01, * *p* < 0.05 versus saline, two-way repeated measures ANOVA followed by Tukey’s post hoc test.

Histamine showed *p* < 0.05 after 1 h, *p* < 0.01 after 3 h and *p* < 0.001 after 5 h as shown in Figure 7. Serotonin displayed *p* < 0.05, *p* < 0.01 and *p* < 0.001 in 1–2, 3–4, and 5 h intervals respectively as displayed in Figure 8, against that of the saline control group. The 2- HF treated group (30 mg kg^−1^) saw a reduction in various stages of inflammation response (initial and final stages) which was activated by a carrageenan sub planter injection at 1–3 h. Likewise, a considerable time-dependent decline in histamine and serotonin induced inflammation was observed with 2-HF administration.

Our tested compound 2-HF at 45 mg kg^−1^ exhibited prominent time-dependent anti-inflammatory potentials shown by a steady decline in the paw volume induced by histamine and the way in which the serotonin model activated an inflammatory response. The aspirin treated group at doses of 50, 100 and 150 mg kg^−1^ showed a significant anti-inflammatory tendency (*p* < 0.05, *p* < 0.01, *p* < 0.001 during 1–5 h) in the carrageenan, histamine and serotonin models of inflammation as compared to the saline control group.

In the xylene-induced ear edema model of inflammation, xylene increased the mass of the ear in the saline control group. The increase in ear edema was considerably decreased by the 2-HF treated group. Our sample 2HF at medium dose considerably reduced ear edema volume when compared with saline.

**Figure 9:**
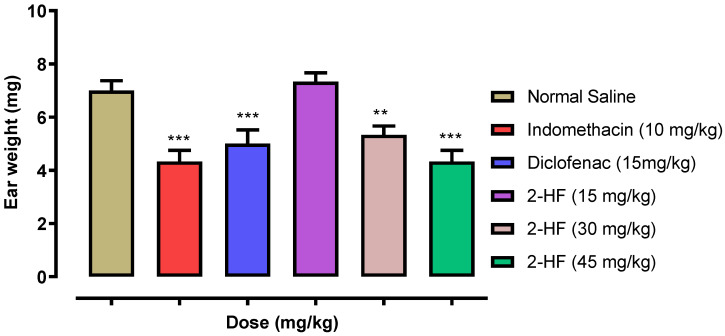
Effect of 2-Hydroxyflavanone, and the standards in xylene-induced ear edema model. Every bar displays the mass of the ear in mg ± SEM. *** *p* < 0.001, ** *p* < 0.01 and * *p* < 0.05 against saline control group. ANOVA one way accompanied by post hoc Dennett’s test was used.

Higher doses of 2-HF caused marked decline (*p* < 0.001) in the mice ear edema. Peripherally acting analgesic agents, including Diclofenac (15 mg kg^−1^) and Indomethacin (10 mg kg^−1^), were utilized as positive controls and demonstrated a significant decline in xylene induced ear edema in mice as compared with saline control group Figure 9**.**

### 2.6. Effect of 2-HF in Neuropathic Pain Model of Cisplatin

#### 2.6.1. Effect of 2-HF in Static Allodynia

**Figure 10:**
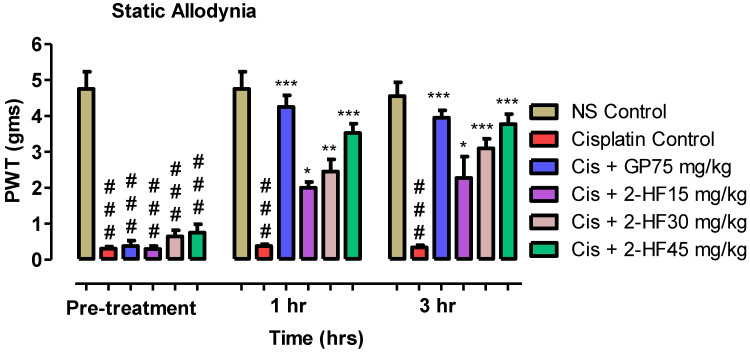
Effect of 2-HF in cisplatin-induced static allodynia. *** *p* < 0.001, ** *p* < 0.01, * *p* < 0.05 for standard drug and cis + 2HF 45 mg kg^−1^, two-way repeated measures ANOVA followed by Tukey’s post hoc test.

Our selected compound was assessed for efficacy in static allodynia with the help of 0.16 to 6 gm von Frey hairs and a specific force for 5 s was applied to the right hind paw (planter surface) by up and down method to evaluate the threshold sensitivity. Gabapentin, a GABA analogue, acts by reducing neuronal excitability and thus declines transmission of pain signals. This is used as a control drug in peripheral neuropathy studies. Combination of standard gabapentin 75 mg kg^−1^ with 2-HF at 15, 30, 45 mg kg^−1^ doses exhibited considerable efficacy as indicated by an increase in paw withdrawal threshold in gm with time (Figure 10). A rise in paw withdrawal threshold in gm was noted for standard GP treated groups as shown in Figure 10.

#### 2.6.2. Cold Allodynia

**Figure 11:**
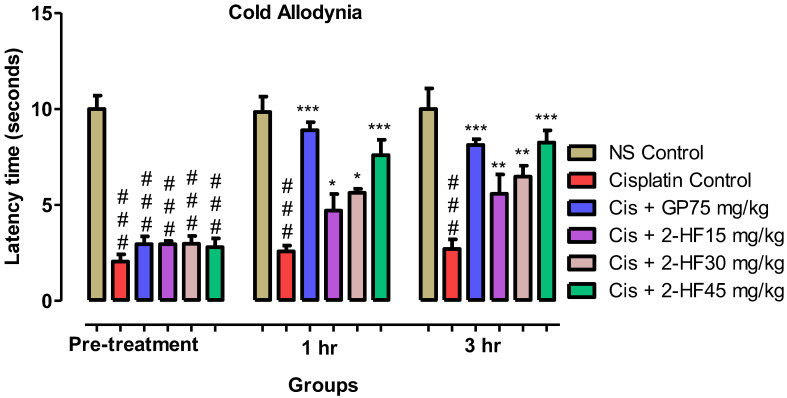
Results of acetone-induced cold allodynia study. Results are mean ± SEM (*n* = 6). *** *p* < 0.001, ** *p* < 0.01, * *p* < 0.05 for standard GP + Cis, two-way repeated measures ANOVA followed by Tukey’s post hoc test.

In comparison to the saline group, paw withdrawal latency in seconds by the application of acetone demonstrated a significant increase after 2-HF administration. Co-administration of 2-HF at doses of 15, 30 and 45 mg kg^−1^ and standard GP at a dose of 75 mg kg^−1^ demonstrated prominent anti-allodynic effects. The paw withdrawal latency was considerably increased in the GP + Cisplatin treated group. Moreover, Cisplatin + 2HF 15 mg kg^−1^ showed an increase in paw withdrawal in a way that was similar to the way in which 2-HF (30 and 45 mg kg^−1^) + Cisplatin displayed a rise in paw withdrawal latency time for 1 and 3 h, as illustrated in Figure 11.

#### 2.6.3. Thermal Hyperalgesia

**Figure 12:**
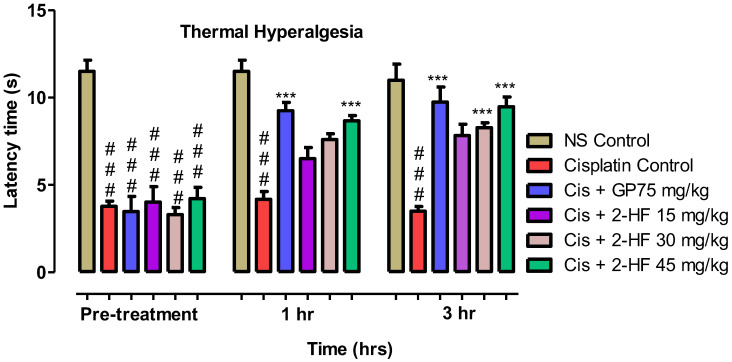
Effect of 2-HF in cisplatin induced neuropathy in hot plate test. *** *p* < 0.001 for GB + Cis and cis + 2-HF at 45 mg kg^−1^, two-way repeated measures ANOVA followed by Tukey’s post hoc test.

A prominent rise in paw withdrawal latency in seconds was noted when cisplatin + 2HF groups were assessed through hot plate analgesiometer for pain threshold. A steady rise in paw withdrawal latency in seconds was noted for GP at 75 mg kg^−1^ + Cisplatin, 2-HF at 30 and 45 mg kg^−1^ for 1 and 3 h. However, the efficacy of compound 2-HF at 15 mg kg^−1^ was non-significant (Figure 12) in comparison to the saline group. Our test compound at 30 mg kg^−1^ combined with cisplatin caused a significant effect during the first and third hours whereas 2-HF at 45 mg kg^−1^ combined with cisplatin exhibited comparable effects during 1–3 h (Figure 12).

#### 2.6.4. Effect of 2-HF on Dynamic Allodynia

**Figure 13:**
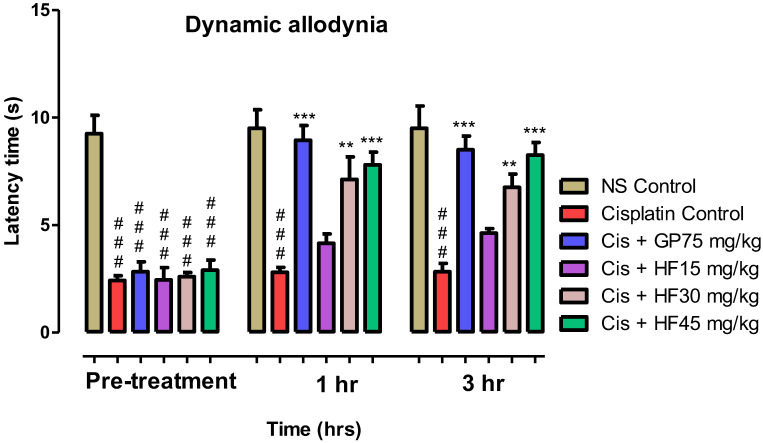
Effect of 2-HF in cisplatin induced dynamic allodynia. Results are shown in mean ± SEM (*n* = 6). There is a significant increase for cisplatin + 2-HF 45 mg kg^−1^ (*** *p* < 0.001, ** *p* < 0.01, * *p* < 0.05), comparable to that of the standard control cisplatin + GP (*** *p* < 0.001), using two-way repeated measures ANOVA followed by Tukey’s post hoc test. Each bar shows mean ± SEM.

A prominent increase in paw withdrawal latency in seconds was noted with 75 mg kg^−1^ GP, cisplatin administered with 45 mg kg^−1^ 2-HF at the 1 and 3 h respectively. Likewise, 2-HF at 30 mg kg^−1^ combined with Cisplatin exhibited a notable rise in paw withdrawal latency (** *p* < 0.01) at 1 and 3 h, respectively, however, for 2-HF (15 mg kg^−1^) + cisplatin no significant increase was noted in paw withdrawal latency after 1 and 3 h treatment as shown in Figure 13.

### 2.7. Acute Toxicity

An acute toxicity test was conducted to determine the safe dosage range of 2-HF. This was achieved by administering doses intraperitoneally to the mice, doubling them each time in steps of 15, 30, 60, 120, 240 and 480 mg/kg. The behavior of the mice was noted for 30 and 60 min and then for 24–72 h. Abnormal behavior such as aggressiveness, cyanosis, spontaneous activity, writhing, ataxia, bizarre behavior and convulsion was observed at higher doses but mortality was not seen, hence, the LD 50 of 2-HF may be higher than 480 mg/kg.

## 3. Discussion

Pain has long been one of the main topics for biomedical researchers, it has particularly attracted many scientists’ attention over the last 5–6 decades around the world [25,26]. In the recent past, flavanones have drawn researchers interest due to their broad pharmacological activities [27], therefore the current project aimed to explore the palliative effects of a flavanone derivative (2-HF) using well established models of neuropathic pain, nociception and inflammation. The statistical results are significant and the compound i.e., 2-HF, was found to be safe up to 480 mg/kg. These results are comparable to standard drugs such as aspirin, tramadol, indomethacin and gabapentin [28].

The anti-nociceptive potential of 2HF (15, 30 and 45 mg/kg) was evaluated in nociceptive models including hot plate test, writhing test and tail immersion model and the results are significant in showing both the central and peripheral anti-nociceptive potentials of 2HF. The anti-nociceptive potential of 2-HF was antagonized by naloxone and PTZ respectively, indicating the opioidergic and GABAergic mechanisms of 2-HF. Moreover, it would have an attraction towards receptors such as mu, kappa and delta. GABAergic agonists may raise the anti-nociceptive potential of morphine, and so may also be therapeutic agents for both acute and chronic pain treatment [29]. Acetic acid-induced writhing test was used for anti-nociceptive action but this model was unable to explain whether the activity was peripheral or central [30]. Prostaglandins, serotonin and bradykinin are released in response to stimulation of the visceral receptors. It also involves nociceptive nerve terminals and peripheral receptors [31]. A significant decline in the writhing number was demonstrated by 2-HF, comparable to that of aspirin. To evaluate the central effects of the 2-HF, hot plate and tail immersion tests were utilized, where an acute nociceptive response was noted upon heat exposure through spinal receptors [32]. Furthermore, 2-HF shows a prominent increase in paw withdrawal latency, measured in seconds, in hot plate test, comparable to that of standard tramadol. Also, 2-HF shows a significant increase in tail latency, measured in seconds, which is controlled by spinal and supra spinal nerves [33].

Previous studies on flavone and its derivatives (methoxy flavone) and flavanone using acetic acid-induced writhing and tail flick methods indicated that the compounds have considerable analgesic potentials [34]. Results reveal that substitution of the methoxyl group at position three of the flavone skeleton considerably reduces analgesic effects, whereas substitution at position five enhances the analgesic potentials of the compounds. Introduction of a methoxy group at positions four and six cause a two-fold increase in the analgesic activity of the compounds. Pre-treatment with naloxone attenuated the analgesic potentials of some derivatives, including 2, 5 and 7 substituted flavones, suggesting an opioid-like mechanism. An important conclusion of this study is that that the flavone skeleton is vital for analgesic activity and the introduction of various groups including hydroxyl and methoxyl causes considerable alteration in the analgesic potentials of the flavone. Double bonds at carbon-2 and carbon-3 of the skeleton are essentials for analgesic activity. The central analgesic mechanism of the compounds is highly reliant on the position of the substitution rather than the type of the substituent [34]. In the current study, our findings are in line with the above conclusions and the involvement of a central nociceptive mechanism might be attributed to the presence of double bonds and substitution at position two of the skeleton.

For the assessment of the anti-inflammatory potentials of 2-HF, four standard animal models of inflammation were used such as carrageenan-, serotonin- and histamine-induced paw edema models and a xylene-induced ear edema model. Carrageenan-induced paw edema is an extensively used approach to determine the anti-inflammatory potentials of test samples. After carrageenan administration, three phases are instigated: the primary, secondary and tertiary phases. The primary phase lasts for 0–1 h and serotonin and histamine is released followed by the secondary phase which lasts for 1.5–2.5 h and where mainly bradykinin is released, while the tertiary phase lasts for 2.5–5 h and mainly prostaglandins are released [35,36]. Our selected compound 2-HF suppressed edema in all the three phases of inflammation as compared to aspirin. Likewise, histamine and serotonin-induced edema were suppressed by 2-HF at the tested doses. Xylene-induced ear edema model is used mostly for the anti-inflammatory activity of non-steroidal and steroidal antiphlogistic agents [37]. Acute neurogenic inflammation occurs because of the increased vascular permeability induced by xylene resulting in ear edema. Our selected compound 2-HF suppressed xylene induced ear edema. Previous anti-inflammatory studies on 2-hydroxyflavanone isolated from *Mimosa pudica* (L.) and its synthetic derivatives indicate that these compounds mediate their therapeutic effect via inhibition of pro-inflammatory cytokines including leukotriene (IL-1β) and Tumor Necrosis Factor Alpha (TNFα) [38]. Dihydroxyflavones have also been reported to exhibit anti-inflammatory potentials [39]. The inflammation is predominantly initiated via the liberation of excessive free radicals in the body which subsequently attack biological macromolecules and initiate a chain reaction [40,41]. Flavonoids and their derivatives are known to have antioxidant potentials and are thus effective in controlling free-radical-induced inflammatory processes. Further, the synergistic anti-inflammatory and analgesic effect of flavonoids and their derivatives might be effective in pain and inflammation. In the current study, the anti-inflammatory potentials coupled with analgesic and anti-neuropathic potentials of 2-HF make it a potential compound for further analysis.

A chemotherapeutic agent, cisplatin, is used extensively to cause neuropathic pain in rodents. In the present study, our selected compound 2-HF was determined in cisplatin-treated groups in standard animal models such as acetone-induced cold allodynia, hot plate test for thermal hyperalgesia, static allodynia via von Frey hairs and dynamic allodynia via cotton ear buds. Cumulatively, our selected compound 2-HF showed a significant effect in all the models of pain.

## 4. Materials and Methods

### 4.1. Chemicals and Apparatus

Digital plethysmometer, Hot-plate-analgesiometer (Harvard Apparatus, MA, USA), Von Frey Filament Kit (US PAT. 5823969. 8512259), Serotonin, Naloxone, Histamine, Xylene, PTZ, Indomethacin, Aspirin and lambda carrageenan were acquired from (Sigma-Aldrich, MO, USA). Glacial acetic acid was acquired from (Pancreac, Spain), Gabapentin from (Lowitt Pharmaceuticals Pvt Peshawar), tramadol from (AGP Pharma, Pakistan), Cisplatin (Pharmedic Laboratories (Pvt) Ltd.), Diclofenac (Indus Pharma, Pakistan). The test compound (2-Hydroxyflavanone) was acquired from Sigma-Aldrich (USA). Other chemicals were acquired from highly regarded vendors. The chemical structure of 2-hydroxyflavanone is given in Figure 1.

### 4.2. Animals and Ethical Approval

Albino mice were utilized in this study and were divided in different groups with 6–8 animals in each group [42]. Standard laboratory water, food and *ad libitum* condition was given to the animals. Water bottles were refilled on a daily basis to avoid any infection. A 22 °C temperature was provided with 12-h dark and 12-h light cycle. All experimental processes were approved by the University of Peshawar ethical committee via letter no: NO 04/EC-18/Phar.

### 4.3. Anti-Nociceptive Studies

#### 4.3.1. Hot Plate Test

About 18–22 g of Albino mice Balb/c were categorized into 5 groups (*n* = 6). Before drugs administration, mice were subjected to initial tests for pre-reading using analgesiometer (Harvard apparatus) preheated to 54 ± 1 °C and mice were removed from the experiment if they did not show response to thermal stimuli, a cut-off time of 30 s was set in order to avoid tissue injury. After half an hour of pre-reading, standard, control, and test samples (15, 30 and 45 mg/kg, respectively) were given intraperitoneally to the label groups, which were then assessed by being placing a over hot plate apparatus. The flinching, paw withdrawal, licking and jumping responses by test animals were recorded post 30, 60 and 90 min of the drugs [43,44].

##### Antagonistic Studies Using Nalaxone and Pentylenetetrazole

Naloxone and pentylenetetrazole at 1 and 15 mg/kg doses, respectively, were given ten minutes prior to the administration of test and standard compounds, to determine their GABAergic and opioidergic mechanisms. The antagonistic effects of naloxone and PTZ were quantified as a higher withdrawal latency of paw, measured in seconds. Naloxone, being an opioid antagonist, antagonizes the anti-nociceptive activities of 2-HF, whereas pentylenetetrazole (PTZ) is a GABA receptor blocker. We administered 1 mg/kg naloxone and 15 mg/kg PTZ to the animals ten minutes before administration of the test (2-HF 30 mg/kg) and standard compounds to determine the involvement of opioidergic and GABAergic mechanisms. Similarly, the antagonistic effects of pentylenetetrazol (15 mg/kg) on the anti-nociceptive effect of 2-HF (30 mg/kg) were observed to assess the potential involvement of GABAergic mechanism for 2-HF.

In a cisplatin-induced peripheral neuropathy model, ne0uropathic pain was induced by cisplatin, and then after induction of pain the 2-HF was administered to determine its efficacy. The efficacy of 2-HF was assessed against static allodynia with the help of 0.16 to 6 gm von Frey hairs. A specific force for was applied to the right hind paw (planter surface) 5 s by up-and-down method to evaluate the threshold sensitivity. Combination of standard gabapentin on paw withdrawal threshold was observed with time. A rise in paw withdrawal threshold in gm was noted for standard gabapentin treated group.

#### 4.3.2. Writhing Test

In this test Albino mice (18–24 g) divided into five groups were utilized. Water and foods were withdrawn from the animals, which were acclimatized for 2 h in the environment. Acetic acid (1%) was injected intra-peritoneally and after five minutes of acetic acid administration writhing behavior was noted for 20 min. Control, standard (aspirin 100 mg kg^−1^) and doses of 15, 30 and 45 mg/kg of the test sample 2-HF were injected 30 min prior to injection of acetic acid and the results were observed [43].

#### 4.3.3. Tail Immersion Model

In this model, albino mice (18–22 g) were used and were classified into five groups. Two hours before the experiment, food and water were withdrawn from the animals and they were adjusted in the experimental condition. Mice were held in an upright position in a restrainer so that their tails were 5 cm below the water level in a water bath preheated to 54 ± 0.5 °C. A cut-off time of 15 s was set for tail flicking and those mice who did not show any response within the cut-off time were excluded from the study. Control, standard (tramadol 30 mg), and test compound 2-HF (15, 30 and 45 mg kg^−1^) were intraperitoneally given to selected groups and their nociceptive effects were noted after 30, 60 and 90 min of drug administration [45].

### 4.4. Anti-Inflammatory Studies

#### 4.4.1. Carrageenan Aroused Paw Edema Model

In the study, albino mice (25 to 30 gm) were utilized and were divided into seven groups. Two hours before starting the experiment food was removed from the animals and they were acclimatized to the experimental condition. Standard (aspirin 50, 100 and 150 mg kg^−1^), and 15, 30 and 45 mg kg^−1^ doses of the test compound (2-HF) and control sample were injected I/P to their label groups 30 min before the sub-planter injection to hind paw of 0.05 mL of freshly prepared 1% carrageenan and inflammation was evaluated with the help of a digital plethysmometer after 0, 1, 2, 3, 4 and 5 h of the carrageenan injection [46].

#### 4.4.2. Histamine-Induced Edema Model

In this model, albino mice both male and female (25–30 gm) were used and classified into seven groups for the standard drug (50, 100, 150 mg kg^−1^), and for doses of 15, 30 and 45 mg kg^−1^ of the test compound (2-HF) and control group. An amount of 0.1 mL of histamine at a dose of 1 mg/mL was injected into the right hind paws of the mice for the induction of edema and the results were calculated with the help of a digital plethysmometer as reported in the carrageenan model previously [43].

#### 4.4.3. Serotonin-Induced Paw Edema Model

Mice, both male and female (25–30 g), were utilized in this test and were divided into seven groups (*n* = 6). Control, standard and 2-HF were intraperitoneally injected into their assigned groups. Serotonin injection (0.001 mL) at the dose of 1 mg/mL was injected into the right hind paw’s sub-planter region and the results were calculated with the help of a digital plethysmometer as reported previously in the carrageenan model [47].

#### 4.4.4. Xylene-Induced Ear Edema Model

In this model, both male and female mice (25–30 gm) were used and arranged into six groups with each group containing six animals. Control drug standards including Diclofenac (15 mg), Indomethacin (10 mg), and test compound 2-HF at doses of 15, 30 and 45 mg were injected intraperitoneally. Edema was induced in the left ear by topically applying xylene (0.03 mL) to the surface (inside and outside) of the left ear whereas the right ear was deemed a control. Post 15 min of the xylene implementation, the mice were assassinated through cervical dislocation and both the ears were amputated and weight and the results measured accordingly [12].

### 4.5. Efficacy in Neuropathic Pain

#### 4.5.1. Cisplatin-Induced Neuropathy

Neuropathic pain caused by chemotherapy is one of the major problems with cancer, it is characterized by burning sensation, tingling, cold allodynia and numbness. This activity was performed to check the effectiveness of 2-HF in the neuropathic pain treatment. Mice with a weight of 20–30 gm were utilized and arranged into six groups.

#### 4.5.2. Induction of Cisplatin-Based Neuropathic Pain

Neuropathic pain was produced in mice by injecting cisplatin (1 mg/Kg/day) intraperitoneally for seven days consecutively and after one hour of the last dose of cisplatin injection, standard and 15, 30 and 45 mg kg^−1^ of test, compound 2-HF were administered. Mice were then evaluated post 1 and 3 h after administration of the standard and test compounds [48].

#### 4.5.3. Cold Allodynia

Mice were arranged into six groups including saline control, disease group, negative control, and positive control GP 75 mg kg^−1^ and 2-HF at the dose of 15, 30 and 45 mg kg^−1^. Mice were kept on an aluminum mesh and acclimatized for 15 min to the environment. Acetone was applied through a syringe or needle to the right hind paw (plantar surface) with a cutoff time of 30 s. Response of licking with respect to time was noted [48].

#### 4.5.4. Mechanical Allodynia or Static Allodynia

Mice were arranged into six groups including saline control, disease group, negative control, and positive control GP 75 mg kg^−1^ and 15, 30 and 45 mg kg^−1^ of 2-HF (test compound). Mechanical Allodynia was assessed by pressing the hind paw with the help of 0.16 to 6 gm von Frey hairs and a specific force was applied to the right hind paw for 5 s (plantar surface) by up-and-down method to evaluate the threshold sensitivity.

#### 4.5.5. Dynamic Allodynia

Mice were arranged into six groups including saline control, disease group, negative control, and positive control GP 75 mg kg^−1^ and 2-HF at doses of 15, 30 and 45 mg kg^−1^. Dynamic allodynia was assessed by striking mildly with cotton swab the middle plantar surface of the left hind paw, cutoff time was set as 15 s. Lifting and licking was assumed as paw withdrawal latency and were noted and calculated [49].

#### 4.5.6. Thermal Hyperalgesia Test

Mice were arranged into six groups including saline control, disease group, negative control, and positive control GP 75 mg kg^−1^ and 2-HF at doses of 15, 30 and 45 mg kg^−1^. Hot plate at 56 °C was maintained and withdrawal response of paw with respect to latency was observed after touching the left hind paw slightly with the plate with 0.5 s as the least value and 10 s as a maximal value as described previously [49].

### 4.6. Acute Toxicity

Acute toxicity test was conducted to assess the safety of the 2-Hydroxyflavanone by giving 2-HF to the mice I/P and doubling the dose each time in steps of 15, 30, 60, 120, 240, 480, with behavior such as aggressiveness, spontaneous activity, cyanosis, writhing, ataxia, bizarre behavior and convulsion assessed as previously reported [50,51].

### 4.7. Statistical Analysis

Graph Pad prism version 5 was utilized to evaluate the data by applying two-way repeated measures ANOVA followed by Tukey’s post hoc test. *p* values < 0.05 were considered statistically significant.

## 5. Conclusions

The selected synthetic flavanone derivative 2-hydroxyflavanone was selected for the purpose of being a new remedy for the treatment of pain. Our findings suggest that 2-HF has significant anti-nociceptive and anti-neuropathic potentials, possibly mediated through GABAergic and opioidergic mechanisms. The test compound showed desirable profile in standard animal pain models as compared with tramadol, gabapentin and aspirin. Hence, it exhibits the capability to be a solution for the management of pain.

## Data Availability

Data related to the current paper can be provided upon request to the corresponding author.
